# Ammonia Gas Sensing Behavior of Tanninsulfonic Acid Doped Polyaniline-TiO_2_ Composite

**DOI:** 10.3390/s151026415

**Published:** 2015-10-16

**Authors:** Venu Gopal Bairi, Shawn E. Bourdo, Nicolas Sacre, Dev Nair, Brian C. Berry, Alexandru S. Biris, Tito Viswanathan

**Affiliations:** 1 Department of chemistry, University of Arkansas at Little Rock, 2801 S. University Ave, Little Rock, AR 72204, USA; E-Mail:bcberry@ualr.edu; 2 Center for Integrative Nanotechnology Sciences, University of Arkansas at Little Rock, 2801 S. University Ave, Little Rock, AR 72204, USA; E-Mails: sxbourdo@ualr.edu (S.E.B.); nicolas.sacre@orange.fr (N.S.); devthenair@gmail.com (D.N.); asbiris@ualr.edu (A.S.B.)

**Keywords:** conducting polymers, composites, chemical properties, electrical properties, ammonia gas sensors

## Abstract

A highly active tannin doped polyaniline-TiO_2_ composite ammonia gas sensor was developed and the mechanism behind the gas sensing activity was reported for the first time. A tanninsulfonic acid doped polyaniline (TANIPANI)-titanium dioxide nanocomposite was synthesized by an *in situ* polymerization of aniline in the presence of tanninsulfonic acid and titanium dioxide nanoparticles. X-ray diffraction and thermogravimetric analysis were utilized to determine the incorporation of TiO_2_ in TANIPANI matrix. UV-Visible and infrared spectroscopy studies provided information about the electronic interactions among tannin, polyaniline, and TiO_2_. Scanning electron microscopy (SEM) along with energy dispersive X-ray spectroscopy (EDS) and atomic force microscopy (AFM) surface analysis techniques were used to investigate the metal oxide dispersions inside polyaniline matrix. Gas sensors were prepared by spin coating solutions of TANIPANI-TiO_2_ and TANIPANI composites onto glass slides. Sensors were tested at three different concentrations (20 ppm, 40 ppm, and 60 ppm) of ammonia gas at ambient temperature conditions by measuring the changes in surface resistivity of the films with respect to time. Ammonia gas sensing plots are presented showing the response values, response times and recovery times. The TANIPANI-TiO_2_ composite exhibited better response and shorter recovery times when compared to TANIPANI control and other polyaniline composites that have been reported in the literature. For the first time a proposed mechanism of gas sensing basing on the polaron band localization and its effects on the gas sensing behavior of polyaniline are reported.

## 1. Introduction

Metal oxides (TiO_2_, ZnO, SnO_2_, MoO_3_, *etc.*) have been utilized as gas sensors over the past several decades, but with some drawbacks, which include inoperability of the sensors at room-temperature conditions (most of them are operative above 200 °C) and long-term instability [1,2,3,4]. Among metal oxides, nanocrystalline TiO_2_ has shown auspicious results in the detection of hydrogen, ammonia, and nitrogen dioxide [3,4]. In addition to the use of metal oxides, a plethora of research has been focused on the development of gas sensors from conducting polymers. Conducting polymers (especially polyaniline, polypyrrole, and polythiophene) have been widely researched as gas sensor materials due to their unique electrical properties and operability at ambient conditions [5,6,7].

Polyaniline (PANI) is one of the many interesting conducting polymers for gas sensing due to its different sensitivities towards multiple gases (NH_3_, CO, H_2_, NO_2_, methanol, H_2_S, hydrazine, *etc.*) [5,6,8,9,10,11,12,13,14,15]. The intrinsic redox and acid/base reactions it can undergo result in different electrical properties that vary easily with doping, making it suitable for gas sensing applications [6,11,16,17,18,19]. PANI can be doped with several acids and these dopants influence the conductivity by changing the electrical, chemical, and structural nature of polymer resulting in active gas sensing sites [20,21]. Potential dopants for PANI include camphor sulfonic acid, *p*-toluene sulfonic acid, hydrochloric acid, sulfuric acid, tanninsulfonic acid, ligninsulfonic acid, *etc.* Among these dopants, tanninsulfonic and ligninsulfonic acid-doped PANI were found to exhibit enhanced chemical stability and thermal degradation properties and have performed as better anti-corrosion materials [22,23,24].

Tannins and lignins are plant based renewable resources abundantly available in nature and can be effectively utilized to complex metal oxides due to the *o*-catechol moieties in their chemical structure [25]. Grafting of tannins and lignins to PANI has already been established in the literature [22,23,24], and in this preparation, tannin is effective in bringing the PANI and metal oxides together. In our previous report, grafting tannin to polyaniline and the interaction of tannins and metal oxides with polyaniline has been discussed in detail [22,23,26]. Electronic properties of PANI are found to be influenced by the presence of metal oxides. Polyaniline-metal oxide composites have been investigated for use in light emitting diodes, rechargeable batteries, anticorrosion coatings, and photovoltaics [6,8,14,19,27].

The combination of PANI and TiO_2_ has resulted in materials with synergistic properties and has aided in the development of new gas sensors. PANI-TiO_2_ composites have been found to overcome drawbacks associated with the individual components due to superior chemical and physical properties [28,29,30,31,32,33,34,35]. Literature studies have identified that 50% TiO_2_-loaded PANI has superior gas sensing activity when compared to other PANI-TiO_2_ compositions. Several studies have explained the superior activity of the composites by this mechanistic approach; doped polyaniline is a hole conductor (p-type) material whereas the TiO_2_ is a good electron acceptor (n-type), when mixed together they can form a p-n junction [28,31,36,37]. The developed p-n junction is sensitive to different gases depending on electron donating/electron accepting nature of the gas. Even though this kind of approach was ideal, it was hard to identify and quantify the generated p-n junction, hence tuning the gas sensing behavior is not a possibility. There is a need for a better approach to identify an exact mechanism for gas sensing, so one can tune the performance of a gas sensor. Current study is focused on elucidating the mechanism for superior activity of TANIPANI-TiO_2_ composite compared to TANIPANI based on the polaron band localization. In addition to polyaniline-metal oxide hybrids, polyaniline-carbon nanotube [38,39] and polyaniline-graphene [40] composites have also been explored for gas sensing applications and have shown improved activity, owing to synergism of the materials compared to individual counterparts. Characterization of polyaniline gas sensors has been performed in several different ways: measuring the change in the surface resistivity [29,34,36,41], current-voltage plots [10,18,28,32,42], and spectroscopy [17]. Among all above-mentioned techniques, measuring the surface resistivity is one of the most facile techniques and has been employed for this study.

Preparation of *ex situ* blends may be easier but *in situ* polymer-metal oxide composites have been found to exhibit stronger interactions between the composite components [22,43]. In the present work, composites of tanninsulfonic acid doped polyaniline-TiO_2_ (TANIPANI-TiO_2_-50%) have been prepared by an *in situ* synthesis technique. Chemical, optical and physical properties of the TANIPANI-TiO_2_ and TANIPANI composites were determined by using several different characterization techniques and the details are discussed at a greater depth. Responsiveness of the sensors for varied concentrations of ammonia gas are explored and a gas sensing mechanism was proposed basing on the polaron band localization studies.

## 2. Experimental Section

### 2.1. Materials

Aniline (Fisher Scientific Company, Fairlawn, OH, USA) was double distilled prior to use. Sulfonated tannin-D3 form (Chevron Phillips, Woodlands, TX, USA) was used as received. A 1M solution of methanesulfonic acid was prepared from 70% methanesulfonic acid (Aldrich Chemicals, St. Louis, MO, USA). Ammonium hydroxide (A.C.S reagent grade) containing 28% to 30% of ammonia was diluted to 1 M solution prior to use. *m*-Cresol (99%), 1-methyl-2-pyrolidinone (NMP) (99%) and sodium persulfate reagent grade (≥98%) (Sigma-Aldrich, St. Louis, MO, USA) were used as received. Isopropyl alcohol (99.5%) (Acros Organics, NJ, USA) was used as received. 10-camphorsulfonic acid (98%) and Titanium Dioxide nano powder (anatase:rutile 80:20, avg particle size 21 nm, 99.5% purity) (Aldrich Chemicals, St. Louis, MO, USA) were used as received.

### 2.2. Synthesis and Processing

Tanninsulfonic acid-doped polyaniline (TANIPANI) was prepared by oxidative polymerization of aniline in the presence of tanninsulfonic acid and methanesulfonic acid using sodium persulfate as oxidant, similar to a method by MacDiarmid *et al.* [44]. A sample of TANIPANI-TiO_2_ with 50% TiO_2_ was synthesized by adding a known amount of TiO_2_ to 2 mL of aniline in the overall reaction mixture. The synthesis was carried out at −10 °C in the presence of isopropyl alcohol in order to prevent the reaction contents from freezing. Details of the synthetic procedure have been published elsewhere [22]. The as synthesized TANIPANI and TANIPANI-TiO_2_ composite was undoped by stirring in 0.1 M NH_4_OH solution, followed by filtration and washing with deionized water, and then stored under vacuum until dry.

### 2.3. Characterization

X-ray diffraction (XRD) studies were performed on powdered undoped TANIPANI-TiO_2_ samples using a Bruker D8-Discover instrument. Cu-Kα line was used as X-ray source operating at 40 kV and 35 mA. The quantity of metal oxides incorporated and thermal stability of samples were evaluated by gravimetric analysis using a Mettler-Toledo TG50 instrument (the samples were heated from 35 °C to 850 °C in alumina crucibles). Absorption spectra of both doped (thin films of polymer-prepared as mentioned in the gas sensor preparation section) and undoped samples (1 mg/mL solutions in NMP) of TANIPANI and TANIPANI-TiO_2_ were evaluated by using a Perkin-Elmer Lamda 19 UV-Vis/near infrared spectrometer. Fourier transform infrared (FTIR) spectra of powder undoped samples were recorded in potassium bromide pellet using a Nicolet Magna-IR 550 spectrometer. Morphology and elemental analysis of TANIPANI-TiO_2_ thin films were analyzed by JOEL 7000F scanning electron microscope equipped with an energy dispersive X-ray spectrometer by EDAX (SEM-EDS). Surface roughness of gas sensors were evaluated using a Veeco-dimension 3100 Atomic Force Microscope (AFM).

## 3. Results and Discussion

**Figure 1:**
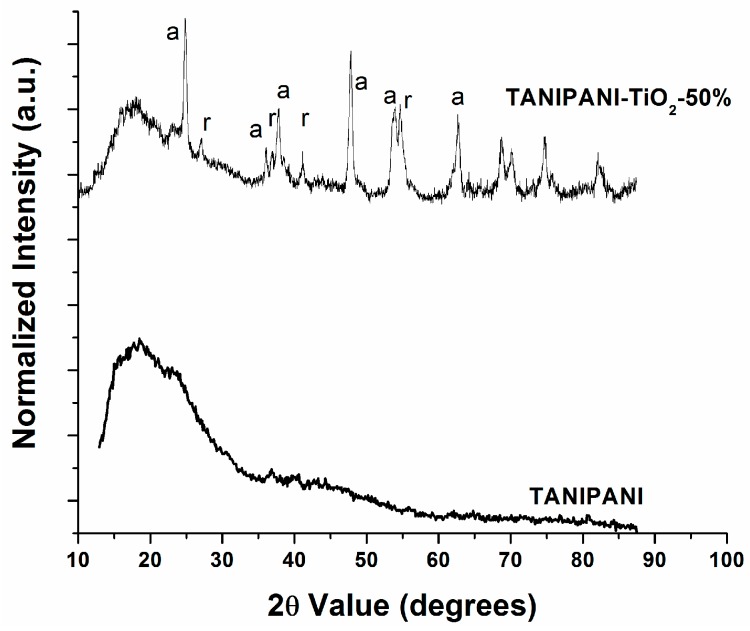
X-ray diffractograms of undoped TANIPANI-TiO_2_ and TANIPANI powders.

In order to determine the incorporation of nano particles in TANIPANI matrix, X-ray diffractometry was employed. This analysis confirmed the presence of TiO_2_ particles within TANIPANI matrix. Even after washing, undoping, and redoping processes, the presence of TiO_2_ in the product suggests a stable composite material with strong interactions between TANPANI and TiO_2_. Both anatase and rutile forms of TiO_2_ were present in the TANIPANI-TiO_2_ composite. The peaks resulting from anatase and rutile are shown in Figure 1, and are designated as “a” and “r”, respectively [22,30,32]. There were three peaks (15°, 18° and 22°) in the samples that arise due to crystalline nature of the polyaniline chains [22,32,45].

**Figure 2:**
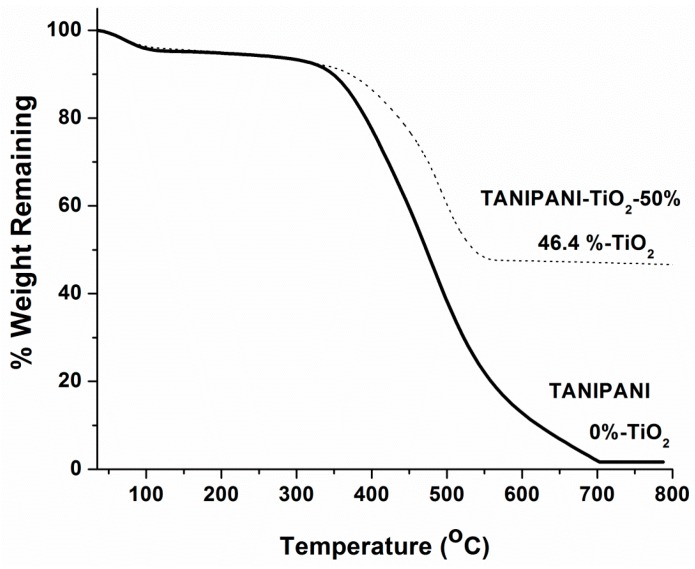
Thermal degradation curves of undoped TANIPANI and TANIPANI-TiO_2_ powder samples.

Thermogravimetric analysis complemented the results obtained by XRD, indicating the presence of metal oxide in TANIPANI-TiO_2_ sample (presented in Figure 2). The presence of metal oxide was evident by the residue in composite material even after heating to 850 °C. The thermal stability of the composite compared to TANIPANI was found to increase by 55.37 °C upon incorporation of TiO_2_. The onset of degradation in TANIPANI was 357.2 °C whereas for the TANIPANI-TiO_2_-50% sample it was found to be 412.57 °C. This also lends support to a strong interaction between the TANIPANI and TiO_2_ leading to a more stable product.

**Figure 3:**
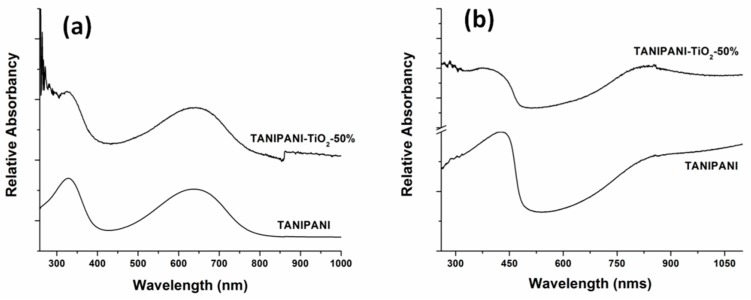
(**a**) UV-Visible absorption spectra of dedoped TANIPANI-TiO_2_ and TANIPANI solutions in NMP solvent; (**b**) UV-Visible absorption spectra of doped TANIPANI-TiO_2_ and TANIPANI thin films spin coated from m-cresol solvent.

Optical spectroscopy has been utilized as a powerful tool for detection of chemical interactions that that occur in PANI composite materials. Doped and undoped forms of polyaniline have characteristic absorption bands in the UV-Visible region. Undoped polyaniline exhibits two unique absorption bands, shown in Figure 3a, one at 640 nm due to the benzenoid to quinoid transition and a second one at 320 nm due to Π-Π^*^ transitions from phenyl rings [17,18,22,29,46,47]. In our previous report [22] we have discussed at a greater length the occurrences of new absorption bands upon incorporation of tannin and metal oxide into polyaniline. Briefly, tannin shows a maximum absorption around 285 nm and TiO_2_ shows a broad range of absorption from 260 nm to 330 nm. In the case of TANIPANI-TiO_2_, there is an additional absorption shoulder at wavelengths lower than 320 nm, which can be attributed to the interaction of tannin and TiO_2_ with polyaniline [22].

Doped polyaniline exhibits two different absorption bands: 420 nm (polaron-Π^*^ transition) and a free carrier tail starting around 600 nm extending into the near-IR (Π-polaron transition) as shown in Figure 3b [17,20,21,22]. In case of the TANIPANI-TiO_2_ sample, three absorption bands were identified; two of these aforementioned bands are attributed to polyaniline and the third band around 280 nm is due to the presence of tannin and TiO_2_ [22]. The bands occurring at 420 nm and 800 nm are blue shifted in the TANIPANI-TiO_2_ sample [29,31,32,43]. Furthermore, the polaron band is more localized (appears like an absorption band) in TANIPANI-TiO_2_ sample, compared to a free-carrier tail that is observed in the TANIPANI. It is also worth noting that the intensity of polaron band is equal to the 420 nm absorption band. Generally, electrons on nitrogen atoms in polyaniline contribute to the polaron delocalization over several benzenoid rings in polyaniline. However, in certain cases when the electrons are not available for long-range delocalization due to poor overlapping of Π-orbitals, only localized polarons are formed. In TANIPANI-TiO_2_ sample, this phenomenon is attributed to the interaction of lone pair electrons on nitrogen atom of polyaniline with empty d-orbital of TiO_2_. It is hypothesized that TiO_2_ functions as a Lewis acid and is not easily displaced from polyaniline.

**Figure 4:**
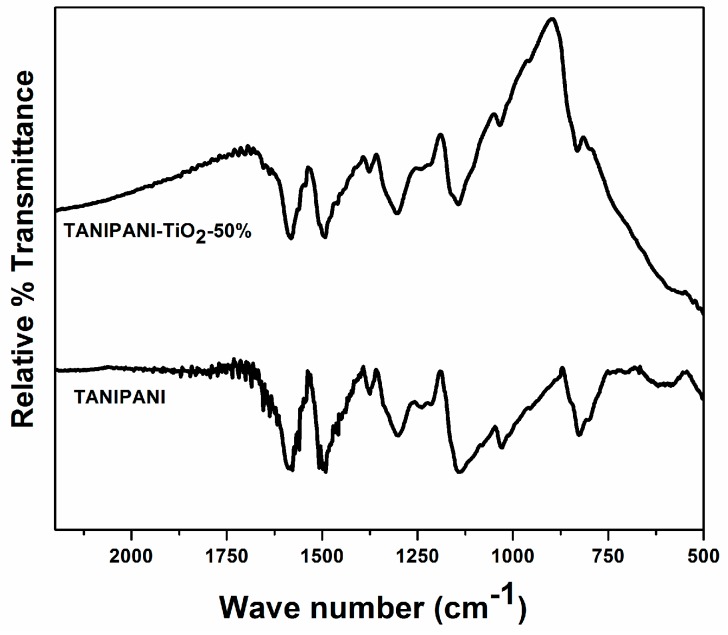
Fourier Transform-Infrared spectra of dedoped TANIPANI-TiO_2_ and TANIPANI powder samples.

Infrared spectral studies provide valuable information about the chemical interaction between polyaniline and TiO_2_. The spectra in Figure 4 shows the occurrence of quinoid C = C and benzenoid C = C stretching vibrations at 1580 and 1490 cm^−1^, respectively, confirming the emeraldine base form of polyaniline [22,34,36,43,47]. The occurrence of sulfonate stretching band at 1030 cm^−1^ band in both the spectra is an indication of tannin sulfonate grafting to polyaniline [22,23]. Titanium dioxide is a broadly absorbing material below 900 cm^−1^ and this absorption peak is clearly evident in TANIPANI-TiO_2_ sample. The interaction of TiO_2_ with polyaniline can be observed from the shift in C-N stretching band of the benzenoid ring from 1301.7 to 1308.2 cm^−1^ and the shift in C-N stretching band of quinoid ring from 1139.7 to 1143.5 cm^−1^ [22,29]. The broadened N-H stretch around 3400 cm^−1^ has been attributed to the interaction of lone pair of electrons on nitrogen of polyaniline with TiO_2_.

**Figure 5:**
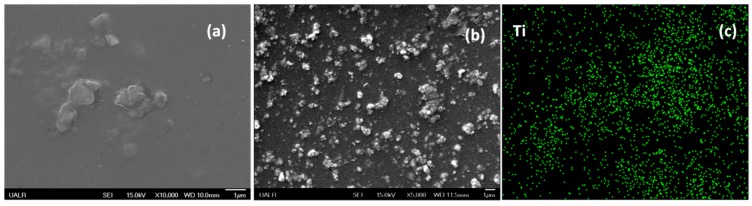
SEM images of (**a**) TANIPANI thin film; (**b**) TANIPANI-TiO_2_ thin film; and (**c**) EDS image showing the dispersion of Titanium in TANIPANI-TiO_2_ thin film.

SEM imaging was performed on thin films of TANIPANI and TANIPANI-TiO_2_ to investigate the morphological features. The morphology of TANIPANI-TiO_2_ was found to be more granular when compared to TANIPANI as shown in Figure 5. Energy dispersive X-ray analysis provided valuable information about the occurrence and dispersion of Titanium in TANIPANI-TiO_2_ thin film sample as shown in Figure 5c. The increase in granular nature of TANIPANI has been attributed to the coating of metal oxide nano particles with TANIPANI.

**Figure 6:**
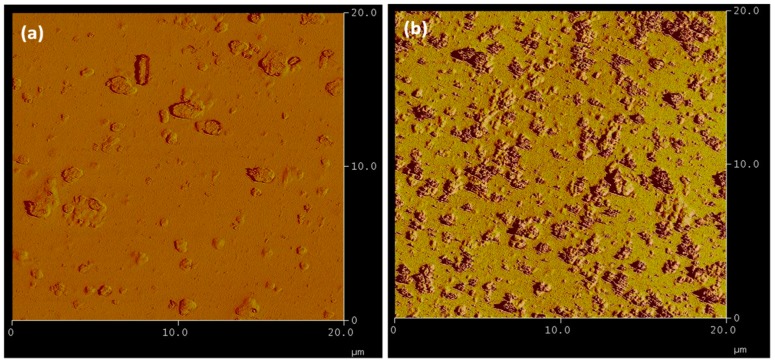
AFM images of (**a**) TANIPANI thin film and (**b**) TANIPANI-TiO_2_ thin film.

A quantitative analysis of the surface roughness of TANIPANI and TANIPANI-TiO_2_ thin films were determined by performing atomic force microscopy studies, images shown in Figure 6. The surface roughness of TANIPANI was found to be 44.51 ± 1.30 nm and TANIPANI-TiO_2_ was found to be 53.12 ± 1.23 nm. The higher surface roughness of TANIPANI-TiO_2_ composite is a contributing factor to the high gas sensing behavior, as it offers more contact points for the ammonia gas detection.

## 4. Ammonia Gas Sensing Application

### 4.1. Gas Sensor Fabrication

**Figure 7:**
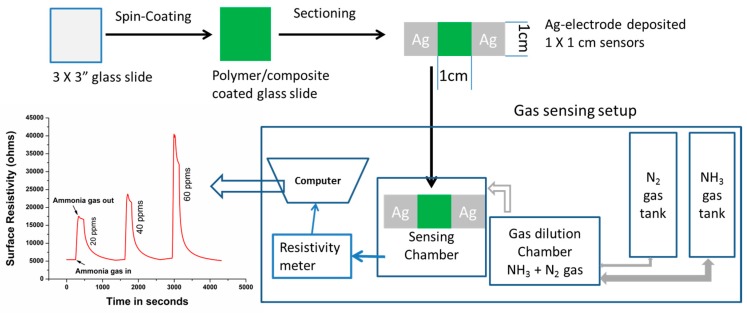
Complete flow chart of the gas sensing process, which includes the sensor fabrication and sensitivity measurement process along with a representative raw sensogram.

In order to promote the formation of thin films with good integrity, camphorsulfonic acid was used as a co-dopant. Specifically, 2% (*w*/*w*) TANIPANI-TiO_2_ and TANIPANI solutions were prepared by mixing a specified amount of undoped sample with camphorsulfonic acid and dissolving the mixture in m-cresol. The weight ratio of CSA to PANI was as follows: for 34.0 mg of polyaniline, 32.0 mg of CSA was used and 3.2 g of m-cresol was added. The mixtures were homogenized using a IKA Ultra Turrax T25 at 20,000 rpm for 15 min, allowed to stir for 1 day, and then centrifuged to remove any undissolved solids. In the case of TANIPANI-TiO_2_-50% composite, the exact amount of polyaniline was determined by omitting the mass of metal oxide from composite using TGA data, and only corresponding amount of CSA is used to prepare solution. The resulting polymer solutions were then spin coated on 3 inch × 3 inch glass slides at 2000 rpm for 2 min. The samples were heated at 70 °C for a period of one hour followed by drying under vacuum. Small specimens were cut from the polymer-coated 3 inch × 3 inch glass slides and used further in preparation of the gas sensors with a 1 cm × 1 cm active area of polymer film between 0.5 cm wide silver electrode strips. The gas sensor fabrication and test setup is depicted in Figure 7.

### 4.2. Gas Sensor Evaluation

Gas sensing measurements were carried out at three different concentrations (20, 40 and 60 ppm) of ammonia using nitrogen as a carrier/diluent gas under ambient conditions (~25 °C). Studies were performed using the nitrogen gas alone to determine any response, but none was observed. The humidity levels in the nitrogen and ammonia gases are <1 ppb of H_2_O, as specified by the gas supplier (Airgas, Little Rock, AR, USA). Nitrogen was used as a diluting gas in order to adjust for different concentrations of ammonia from a 200 ppm ammonia gas cylinder (balance nitrogen) according to the following formula:

(1) $$Final\;NH_{3}\;Gas\;Conc.\;\left(ppm\right)\;=\;\frac{Pressure\;\left(NH_{3}\right)}{Total\;Pressure\;\left(NH_{3}+\;N_{2}\right)}\;\times \;Initial\;Conc.\;of\;NH_{3}\left(ppm\right)$$

In order to dilute the ammonia to appropriate levels, a mixing chamber was utilized—an appropriate amount of 200 ppm NH_3_/N_2_ was introduced followed by the pure nitrogen to arrive at the desired concentrations. Once the correct amount of gases were introduced to the mixing chamber, this diluted ammonia gas was vented into the gas testing chamber where the sensor had been placed under vacuum conditions. In order to measure the film resistivity, a Keithley 2400 source meter was connected to the gas sensor using wires terminated with alligator clips. Our design is such that all gas is introduced at one time to the sensor and its response recorded for 60 s; after 60 s, the chamber is vented. The venting of the chamber takes around 2 min. The sensor is immediately brought to ambient conditions once the ammonia is completely vented. The sensor is allowed to come back to a consistent baseline resistivity. Then the same procedure is repeated for next higher concentration of the gas. During the gas venting step, there is a small signal recovery as can be seen in Figure 7, the sudden drop in the resistivity takes place once the sensor is exposed to ambient conditions. The changes in film resistivity were monitored at one second intervals as follows: (1) sensors were initially exposed to 20 ppm ammonia gas, after which the ammonia gas was vented and the sensor was brought to ambient conditions; (2) sensors were then allowed to return to their initial resistivity values; and (3) Steps 1 and 2 were repeated using higher concentrations of ammonia. Gas sensing measurements were repeated three times for each concentration of ammonia; the response value, response times, and recovery times are presented as an average (with standard deviation) of the three runs. Figure 7 provides a complete schematic of the gas sensing testing process.

### 4.3. Ammonia Gas Sensing Results and Discussion

The physico-chemical properties of polyaniline are altered due to the dynamic doping-undoping processes. These changes are manifest in electrical resistivity when polyaniline is exposed to acidic (low resistivity) and basic (high resistivity) environments. Therefore, an increase in resistivity of a polyaniline-based gas sensor occurs when exposed to different concentrations of ammonia gas. The effects of ammonia on the resistivity of TANIPANI gas sensors are shown in Figure 7. Resistivity of all samples increased with increase in concentration of the ammonia gas from 20 ppm to 60 ppm.

**Figure 8:**
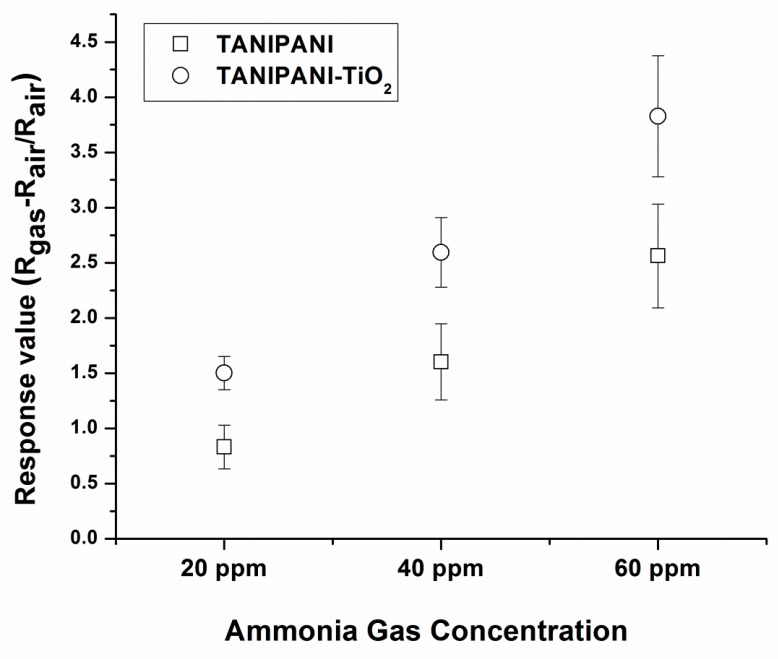
Response values of TANIPANI and TANIPANI-TiO_2_ when exposed to 20 ppm, 40 ppm and 60 ppm of ammonia gas for a period of 60 s.

While the increase in concentration of ammonia gas corresponded to an increase in resistivity, in order to better assess the results, response values, response times, and recovery times were determined. Response values were calculated using the following formula,

(2) $$Response\;Value=\frac{Resistivity\;\left(ammonia\right)-\;Resistivity\;\left(air\right)}{Resistivity\;\left(air\right)}$$

where the resistivity values are determined in a test gas (ammonia in this case) and in air (ambient environment) [21,34,36,37,42]. Response values were found to be higher in case of TANIPANI-TiO_2_ when compared to TANIPANI alone. The data of response values is shown in Figure 8. Both the TANIPANI and TANIPANI-TiO_2_ sample showed an increase in response with increase in concentration of the ammonia gas.

**Scheme 1:**
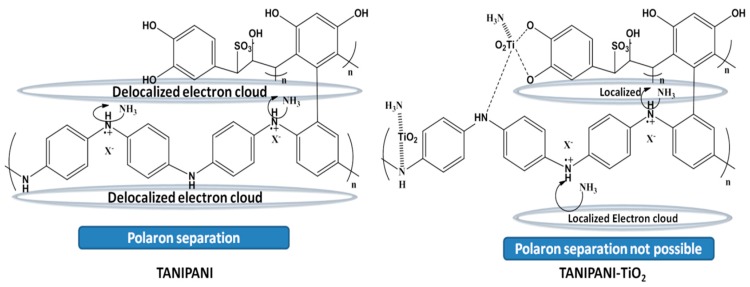
TANIPANI and TANIPANI-TiO_2_ ammonia gas sensing mechanism basing on the polaron separation and electron delocalization.

The response value of TANIPANI-TiO_2_ was found to be 60% higher than that of TANIPANI. Incorporation of TiO_2_ into TANIPANI matrix has a profound effect on the gas sensing ability. In the previously discussed UV-Visible spectral studies, formation of a localized polaron band was observed in TANIPANI-TiO_2_. This can be attributed to the coordination of TiO_2_ with the amine nitrogen of polyaniline. The polarons formed during the doping process on the imine nitrogens are not readily separated, resulting in these aforementioned localized polarons. From a gas sensing perspective, the result is a more pronounced response for the TANIPANI-TiO_2_ composite due to a more energetically favorable dedoping when exposed to ammonia. In addition to the localized polarons, the interaction of TiO_2_ with polyaniline results in an electron withdrawing effect (inductive) on a section of the polymer backbone. Therefore, the surrounding protons become much more acidic and reactive towards ammonia and react readily with the lone pair of electrons on ammonia molecule with subsequent dedoping of polyaniline chain. As a result of this process, conductivity decreases due to the removal of proton from polyaniline. Higher response of TANIPANI-TiO_2_ composite compared to TANIPANI is due to the localized polaron band of the polyaniline chain, which is more easily disrupted by ammonia, hence the dedoping is more efficient. Whereas, with TANIPANI it is hard to disrupt the delocalized polaron band and dedoping is not readily favored. Scheme 1 shows the proposed mechanism of gas sensing activity based on the polaron band localization. Moreover, the surface roughness (Figure 6) of the TANIPANI-TiO_2_ is higher compared to TANIPANI offering more contact points for the ammonia gas to occupy.

**Figure 9:**
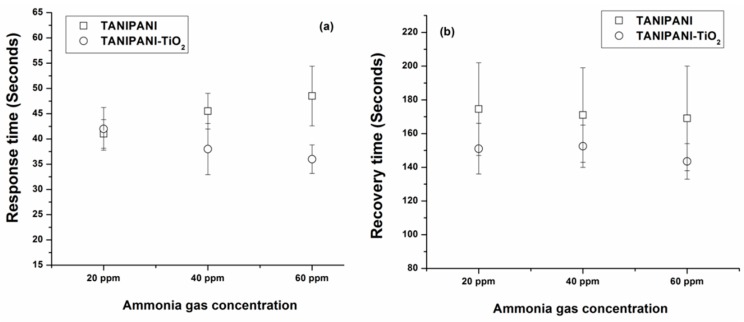
(**a**) Response times of TANIPANI-TiO_2_ sensors after exposure to 20 ppm, 40 ppm and 60 ppm of ammonia gas; (**b**) Recovery times of TANIPANI-TiO_2_ samples after withdrawing 20 ppm, 40 ppm and 60 ppm of ammonia gas.

Response time is defined as the time required for the sensor to change its resistivity value by 90% after the exposure to ammonia gas. Figure 9a shows the response times of TANIPANI and TANIPANI-TiO_2_ at different concentrations of ammonia gas. The response times are shorter for the TANIPANI-TiO_2_ sample compared to TANIPANI alone, indicating a superior performance of the metal oxide loaded polymer sensor. A decrease in response times for the metal oxide loaded sample can be attributed to the favorable dedoping due to the formation of localized polaron band in the presence of metal oxides and higher surface roughness. Recovery time is defined as the time that is required for the sensor to return to 90% of initial resistivity value after removal of ammonia gas from the testing chamber. The recovery times of TANIPANI-TiO_2_ after the removal of ammonia gas was found to be better than that of TANIPANI alone, as shown in Figure 9b. Little or no drift was observed in the baseline resistivity values of the sensors during the measurements with different concentrations of ammonia gas.

The overall response values of the TANIPANI-TiO_2_ sample were found to be superior to any other PANI-TiO_2_ samples in the literature [16,20,21,29,31,34,36,41,43]. Table 1 represents the summary of literature studies on PANI-TiO_2_ as compared to the current work. The higher activity of TANIPANI-TiO_2_ sensor compared to other studies can be attributed to metal oxide complexation by tanninsulfonic acid-doped polyaniline and the electron transfer reactions that take place between ammonia gas and polyaniline in presence of metal oxides.

**Table 1:** Response values of some polyaniline and polyaniline-TiO_2_ composites available in literature.

Samples	Response Value (20 ppm)	Response Value (40 ppm)	Response Value (60 ppm)	Reference
PANI/TiO_2_	0.12	0.29	0.375	30
PANI/TiO_2_	1.67 (23ppm)	2.33 (47 ppm)	N/A	32
PANI/TiO_2_	1.33	2.80	N/A	29
PANI/TiO_2_	0.22	0.40	0.65	18
TANIPANI/TiO_2_	1.50	2.59	3.82	Current work
PANI	0.49	0.74	N/A	32
PANI	N/A	0.83	0.88	17
TANIPANI	0.83	1.60	2.56	Current work

Polyaniline-metal oxide based gas sensors for detecting various gases have been explored by many groups and these studies have identified that PANI-metal oxides are highly selective to ammonia [16,21,48]. While the sensors in this study were tested solely for ammonia response, it is expected that the sensors will also respond to a variety of basic environments (proton acceptors). However, ammonia analogues, such as mono-, di-, and tri-substituted amines would be detected by the sensors, albeit with varying degrees of selectivity due to the electronic inductive and steric nature of the basic molecules [16,48]. The influence of humidity on the sensor resistance is negligible compared to the sensitivity of ammonia gas [16,41].

## 5. Conclusions

A TANIPANI-TiO_2_ composite was prepared by a facile *in situ* synthesis and was shown to be effective at sensing ammonia gas. The thermal stability of the metal-oxide polymer composite was significantly higher than the TANIPANI alone. Interactions among the metal oxide, tannin, and polyaniline are evident from spectroscopic analysis. A strong interaction of metal oxide with polyaniline and formation of localized polaron band has been determined to assist in doping and dedoping processes of TANIPANI chain which leads to faster response times and better ammonia gas sensing characteristics for the TANIPANI-TiO_2_ composite. The TANIPANI-TiO_2_ composite can be considered superior to other composites in the literature, due to the higher sensitivity of the composite to ammonia gas. One can easily vary the amount of dopant and metal oxides and tune the polaron band, this way a superior composite material can be synthesized for gas sensing application.
